# Prevalence and risk factors of malaria among children in southern highland Rwanda

**DOI:** 10.1186/1475-2875-10-134

**Published:** 2011-05-18

**Authors:** Jean-Bosco Gahutu, Christian Steininger, Cyprien Shyirambere, Irene Zeile, Neniling Cwinya-Ay, Ina Danquah, Christoph H Larsen, Teunis A Eggelte, Aline Uwimana, Corine Karema, Andre Musemakweri, Gundel Harms, Frank P Mockenhaupt

**Affiliations:** 1 Butare University Teaching Hospital, Faculty of Medicine, National University of Rwanda, Butare, Rwanda; 2 Institute of Tropical Medicine and International Health, Charité-University Medicine Berlin, Berlin, Germany; 3 German Development Cooperation (GIZ), Health Programme, Kigali, Rwanda; 4 Division of Infectious Diseases, Tropical Medicine and AIDS, Academic Medical Centre, Amsterdam, The Netherlands; 5 National Malaria Control Programme/Malaria Unit, TRAC Plus, Kigali, Rwanda

## Abstract

**Background:**

Increased control has produced remarkable reductions of malaria in some parts of sub-Saharan Africa, including Rwanda. In the southern highlands, near the district capital of Butare (altitude, 1,768 m), a combined community-and facility-based survey on *Plasmodium *infection was conducted early in 2010.

**Methods:**

A total of 749 children below five years of age were examined including 545 randomly selected from 24 villages, 103 attending the health centre in charge, and 101 at the referral district hospital. Clinical, parasitological, haematological, and socio-economic data were collected.

**Results:**

*Plasmodium falciparum *infection (mean multiplicity, 2.08) was identified by microscopy and PCR in 11.7% and 16.7%, respectively; 5.5% of the children had malaria. PCR-based *P. falciparum *prevalence ranged between 0 and 38.5% in the villages, and was 21.4% in the health centre, and 14.9% in the hospital. Independent predictors of infection included increasing age, low mid-upper arm circumference, absence of several household assets, reported recent intake of artemether-lumefantrine, and chloroquine in plasma, measured by ELISA. Self-reported bed net use (58%) reduced infection only in univariate analysis. In the communities, most infections were seemingly asymptomatic but anaemia was observed in 82% and 28% of children with and without parasitaemia, respectively, the effect increasing with parasite density, and significant also for submicroscopic infections.

**Conclusions:**

*Plasmodium falciparum *infection in the highlands surrounding Butare, Rwanda, is seen in one out of six children under five years of age. The abundance of seemingly asymptomatic infections in the community forms a reservoir for transmission in this epidemic-prone area. Risk factors suggestive of low socio-economic status and insufficient effectiveness of self-reported bed net use refer to areas of improvable intervention.

## Background

Recent years have seen a substantial increase in malaria control activities. Particularly in East Africa, growing evidence suggests a decline in malaria transmission, morbidity and mortality over the last decade [1-5]. Control measures considered vital to this improvement are the deployment of artemisinin-based combination treatment (ACT), distribution of long-lasting insecticide-treated nets (LLINs), and indoor residual spraying [3,6].

Rwanda is a prime example for the impact malaria control can have. Since 2000, several million insecticide treated nets (ITNs) have been distributed (mostly LLINs) increasing the percentage of the population (10 million) covered by nets to potentially ≥70%. In parallel, ACTs have been dispensed on a large scale. In 2007, 56% of households were considered to own a net and 56% of children to sleep under one [4]. Surveillance and health facility based data indicate that by 2007-2008 these efforts were associated with approximately 50% or higher declines in confirmed outpatient cases, inpatient cases, and deaths due to malaria in children <5 years old [4,7].

While this progress does not appear to be questionable, the extent of the declines as deduced from facility-based data might differ at community level. For instance, community-level case management programmes [8] have been reported to shift primary treatment from health centres to villages and thus decrease the health-facility burden [9]. Such a trend, however, does not necessarily reflect the situation in the community [10,11].

One aim of the present study was therefore to provide up-to-date malariologic data at the levels of community, health centre, and district hospital for a highland area in southern Rwanda from where no published material exists so far. In addition, the study aimed at identifying (modifiable) factors associated with *Plasmodium *infection and malaria in this population.

## Methods

### Study area and sampling

**Figure 1:**
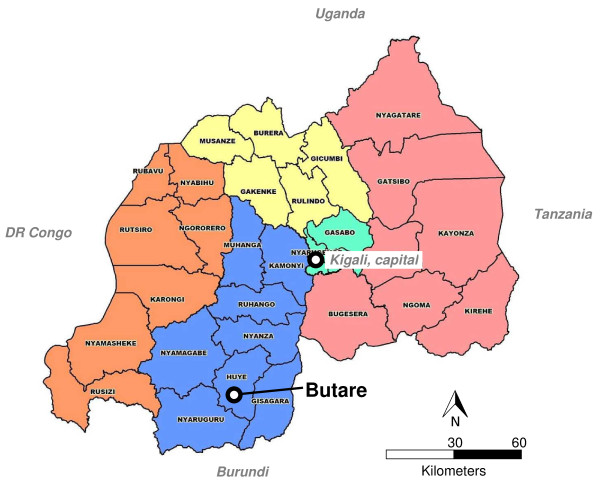
**Rwanda administrative map, with Butare location**. Map displaying the five provinces of Rwanda (Northern, Eastern, Southern, Western, Kigali) and district boundaries.

Butare (population approximately 100,000; altitude 1,768 m) is the capital of Huye district, southern province of Rwanda. Located on the central plateau of Rwanda (Figure 1; average altitude, 1,700 m; yearly rainfall, 1,200 mm; mean temperature, 19°C), Butare is surrounded by densely populated farmland hills. Despite two rainy seasons (October-November; March-May), the area is prone to drought. The present study was conducted from January 18 to March 26, 2010 but the rainy season started as early as late January in this year.

Rwanda has a mandatory health insurance system in which the mutual health insurance scheme *(mutuelle de santé *) is the most widespread. At an annual cost of 1,000 Rwandan Francs (1.28 €; February 2010) per capita, treatment of common diseases is basically free of charge including utilization of district and provincial hospitals provided there is adherence to a strict referral system [12]. Governmental health services in Butare area are provided by several primary health centres, Kabutare district hospital and Butare University Teaching Hospital (CHUB, *Centre Hospitalier Universitaire de Butare *).

**Figure 2:**
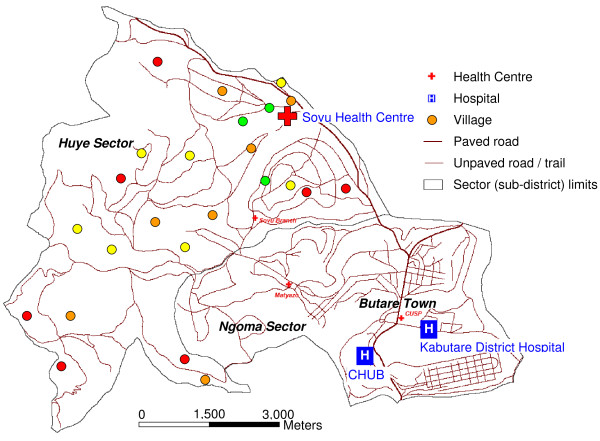
**Study area, Butare town and Huye sector**. Community children were recruited in 24 villages in the rural Huye sector (subdistrict), and patients at Sovu Health Centre and Kabutare district hospital. The prevalence of *P. falciparum *infection (PCR) among children under five years of age in the villages (sample sizes, 18-26) is marked by colour: green, < 5%; yellow, 5 ≤ 10%; orange, 10 ≤ 20%; red, >20%.

The study was designed as a cross-sectional survey to assess the prevalences of malaria, HIV, and soil-transmitted helminths in children under five years of age in the CHUB catchment area, i.e. at the levels of community, health centre, and district hospital. The present report focuses on the malaria situation. For the community level, the neighbouring rural Huye subdistrict (*sector *; population approximately 20,000) was chosen (Figure 2). Based on most recent census data, each 25 households were randomly chosen in a total of 24 randomly selected villages. Community health workers visited these households, randomly selected one child per family, and asked the child to be presented to the study team located at Sovu health centre (or a non-permanently staffed branch) on a scheduled (usually next) day. Thereby, balanced recruitment into the age strata <1, 1 < 2, 2 < 3, 3 < 4, and 4 < 5 years was aimed at. In parallel, ≥100 paediatric patients aged five years or less and presenting at the primary Sovu health centre and at the referral Kabutare district hospital, i.e. the health facilities serving this population, were successively recruited. All children's parents were thoroughly informed on the purpose and procedures of the study, and recruitment was preceded by HIV pre-counselling and obtaining informed written consent. The study was reviewed and approved by the National Ethics Committee, Republic of Rwanda.

### Examinations

Brief questionnaires were filled in on socio-economic aspects of the children's families including household assets; specification of bed nets, e.g. impregnated or not, was omitted. All children were examined by a physician, a medical history obtained, and a venous blood sample collected. Age, sex, weight, height, mid-upper arm circumference (MUAC), and fever (axillary temperature ≥37.5°C) were documented. Haemoglobin (Hb) levels were measured by a HemoCue photometer (Angelholm, Sweden). Anaemia was defined as an Hb level < 11 g/dL. Intestinal parasites were screened for by direct wet mount microscopical stool examination, and urinary tract infection by dipstick (Multistix 10 SG, Bayer, Germany). Malaria parasites were counted *per *200 white blood cells (WBCs) on Giemsa-stained thick blood films, while the patient was waiting. Children with malaria parasites were treated with artemether-lumefantrine. Other diseases were treated according to Rwanda health authority guidelines [13]. Following duplicate readings *per *200 WBCs at the CHUB central laboratory and the Institute of Tropical Medicine & International Health in Berlin, the definite parasite density was calculated on the basis of a putative mean WBC count of 8,000/μL. These data were used for analysis. Malaria was defined as any microscopically visible parasitaemia *plus *fever or a history of fever within the preceding 48 hours. DNA was extracted (Qiamp blood kit; Qiagen, Germany), and *Plasmodium *species and submicroscopic infections were identified by semi-nested multiplex polymerase chain reaction (PCR) assays [14]. For all PCR positive samples, sequences corresponding to the allelic families of the *Plasmodium falciparum *merozoite surface protein 1 (*msp1 *) block 2 (K1, Mad20, Ro33) and of *msp2 *block 3 (FC27, IC) were amplified in five separate PCR assays [15]. Size variation within the alleles can be used to discriminate different parasite clones by PCR fragment length polymorphism, visualised on 3% GTG^® ^-agarose gels (Biozym, Germany) and analysed using GeneSnap software (SynGene, UK). In case of negative or inconclusive PCR results, assays were repeated maximally twice. Multiplicity of infection (MOI) was calculated as the highest number of fragments for either *msp1 *or *msp2*. Residual (pre-treatment) concentrations of chloroquine and pyrimethamine (indicative of sulphadoxine-pyrimethamine) in plasma were determined by ELISA with limits of detection of 5 ng/mL and 10 ng/mL, respectively [16].

### Statistical analysis

Data analysis was performed using Statview 5.0 (SAS Institute Inc.). Continuous variables were compared between groups by the non-parametric Mann-Whitney or Kruskal-Wallis tests, and proportions by χ^2 ^test or Fisher's exact test. Odds ratios (ORs) and 95% confidence intervals (95% CIs) were computed. Non-parametric ordinal regression analysis was performed to assess significantly differential effects of independent factors, e.g. infection, on a dependent variable, e.g. Hb levels, between groups. Despite non-parametric comparisons and for tangibility, parasite densities and Hb concentrations are displayed as geometric mean parasite densities (GMPDs) and means, respectively. Evaluation of determinants of *P. falciparum *infection and malaria was performed by logistic regression analysis. Stepwise backward selection was performed, and final models included those factors that retained statistical significance. A p-value < 0.05 was considered statistically significant.

## Results

### Study participants

A total of 749 children were examined including 545 from the rural Huye communities, 103 from Sovu health centre, and 101 from Kabutare district hospital (Table 1). As compared to community children, those attending the health centre or the district hospital were slightly younger. In the communities, fever and a history of fever within the preceding 48 hours were rare (3% and 10%) but common in the health centre (35%, 70%) and in the district hospital (27%, 48%). The primary clinical diagnoses differed between the groups (overall, *P *< 0.0001). In community children, the leading one was gastro-intestinal affection including gastroenteritis, amoebiasis and helminthiasis. In health centre and hospital, the leading primary diagnosis was respiratory tract infection (Table 1). Chloroquine in plasma was found in 3.7% (28/747) of all children at a median concentration of 15 ng/mL (range, 8-240). Only one child exhibited pyrimethamine in plasma (50 ng/mL).

**Table 1:** Characteristics of 749 children from southern highland Rwanda

Parameter	Huye communities	Sovu Health Centre	Kabutare District Hospital	*P *
No. (%)	545 (72.8)	103 (13.8)	101 (13.5)	
Age (months)	31.1 (1-60)	28.3 (1-59)	27.2 (1-60)*	0.03
Proportion girls (%)	45.5	50.5	50.5	0.48
Weight (kg)	11.3 (3.5-18.8)	10.9 (3.8-20.0)	11.0 (3.3-19.0)	0.24
Height (cm)^a ^	80.0 (41-108)	76.1 (42-112)*	78.5 (52-110)	0.02
MUAC (cm)^b ^	13.7 (5.0-18.0)	14.2 (10.5-19.0)*	13.6 (8.0-18.5)†	0.04
Axillary temperature (°C)^c ^	36.7 (36.0-40.6)	37.4 (36.0-40.1)*	37.0 (35.8-39.4)†	<0.0001
Fever (%)	3.3 (18/543)	35.0*	26.7*	<0.0001
History of fever, last 2 days (%)	9.8 (49/502)	69.4 (68/98)*	47.5 (47/99)*†	<0.0001
Hb (g/dL)^b ^	11.3 (1.7-15.3)	11.4 (1.4-16.6)	11.1 (4.3-16.8)	0.89
Anaemia (Hb < 11 g/dL),%	34.1	35.0	32.0 (32/100)	0.89
Severe Anaemia (Hb<7 g/dl),%	1.8	1.9	7.0 (7/100)*	0.01
Primary diagnosis on examination^d ^				
Healthy child	25.4	4.3*	1.5*	<0.0001
Gastro-intestinal tract affection^e ^	31.9	25.0	19.4*	0.04
Respiratory tract infection	7.3	34.3*	26.1*	<0.0001
Malaria (suspected)^f ^	9.7	19.3*	11.9	0.0001
Severe malnutrition (clinically)	8.2	1.4*	3.7	0.01
Skin infection	4.6	5.7	4.5	0.67
Burns, wounds, accidents, etc.	2.1	2.1	14.9*†	<0.0001
Severe anaemia (clinically)	2.4	0.7	3.0	0.41
Conjunctivitis	1.4	1.4	3.7	0.11
Disability	1.1	0	5.2*†	<0.0001
Oral problems	0.9	1.4	3.7*	0.02
Urinary tract infection	0.6	2.9*	2.2	0.02
Others, missing data	4.4	1.4	0*	0.03

Numerical data are means (range) unless otherwise indicated, and compared by the non-parametric Kruskal Wallis or Mann Whitney U tests. Proportions were compared by χ^2 ^test or Fisher's exact test. MUAC, mid upper arm circumference; Hb, haemoglobin; GMPD, geometric mean parasite density; Malaria, definition ^a ^, *n *= 746; ^b ^, *n *= 748; ^c ^, *n *= 747; ^d ^, as judged by study physician, several diagnoses per child possible; No. of diagnoses: community, *n *= 658; health centre, *n *= 134; hospital, *n *= 140. ^e ^, includes gastroenteritis, amoebiasis, helminthiasis, and others; ^f ^, based on field-based microscopy and clinical judgement. *, difference to Huye communities, *P *< 0.05; †, difference to Sovu health centre, *P *< 0.05

The three groups showed large differences in the socio-economic characteristics of the children's families (Table 2). In the communities, most children lived in rural areas, the average monthly family income was low; one third of the parents had no education at all, and almost all worked as farmers or labourers. Accordingly, asset ownership was generally limited. Less than half of the children were covered by any health insurance; for slightly more than half, a bed net was reported to have been used in the preceding night. Compared to that, socio-economic parameters almost consistently indicated better conditions among children attending the health facilities (Table 2). In particular, among health facility attendees, 85% were covered by a health insurance, and the rate of self-reported bed net use was 71%. Many socio-economic parameters were inter-related. Monthly family income, for instance, was higher in those with a health insurance than in those without (medians, 10,000 *vs*. 5,000 Rwandan Francs, *P *< 0.0001) and higher in those using bed nets as compared to non-users (8,000 *vs*. 5,000 Rwandan Francs, *P *= 0.0003).

**Table 2:** Selected socio-economic characteristics in 749 children from southern highland Rwanda

Parameter	Huye communities	Sovu Health Centre	Kabutare District Hospital	*P *
No.	545	103	101	
Rural residence (%)	95.1 (507/533)	73.7 (70/95)*	66.0 (66/100) *	<0.0001
Monthly family income (RwF)^a ^	9124 (0-100,000)	31,505 (0-300,000) *	28,916 (500-350,000) *	<0.0001
Mothers education (%)				
None	30.4 (165/543)	20.4 (21/103)	14.9 (15/101)	
Primary	67.0 (364/543)	68.0 (70/103)	69.3 (70/101)	
Secondary or higher	2.6 (14/543)	11.7 (12/103) *	15.8 (16/101) *	<0.0001
Mother's occupation farmer/labourer (%)	98.7 (533/540)	92.2 (95/103) *	78.2 (79/101) *†	<0.0001
Father's education (%)				
None	36.5 (195/534)	19.6 (20/102)	19.8 (20/101)	
Primary	60.1 (321/534)	67.6 (69/102)	60.4 (61/101)	
Secondary/tertiary	3.4 (18/534)	12.7 (13/102) *	19.8 (20/101) *	<0.0001
Father's occupation (%)				
Farmer/labourer	86.9 (472/543)	73.3 (74/101)	65.0 (65/100)	
Else	5.5 (30/543)	25.7 (26/101)	35.0 (35/100)	
Died/left/prisoner	7.6 (41/543)	1.0 (1/101) *	0 *	<0.0001
No. of people/household^a ^	5.5 (2-12)	5.2 (3-12)	5.0 (2-12) *	0.03
No. of siblings^a ^	2.0 (0-9)	1.8 (0-7)	1.4 (0-6) *	0.002
Household asset present (%)				
Electricity	1.3 (7/542)	11.7 (12/103) *	23.8 (24/101) *†	<0.0001
Piped water	14.3 (77/540)	6.8 (7/103) *	37.6 (38/101) *†	<0.0001
Radio	43.2 (233/539)	67.0 (69/103) *	77.2 (78/101) *	<0.0001
TV	0.7 (4/541)	5.9 (6/102) *	12.9 (13/101) *	<0.0001
Cupboard	8.9 (48/540)	24.3 (25/103) *	37.6 (38/101) *†	<0.0001
Bicycle	9.1 (49/540)	37.9 (39/103) *	27.7 (28/101) *	<0.0001
Motor-bike	0.6 (3/540)	2.9 (3/102)	1.0 (1/101)	0.07
Fridge	0 (0/541)	1.0 (1/102) *	2.0 (2/101) *	0.01
Cattle	13.0 (70/539)	43.7 (45/103) *	20.8 (21/102) *†	<0.0001
Health insurance present (%)^c ^	43.0 (234/544)	90.2 (92/102) *	79.3 (80/101) *†	<0.0001
Child received any drug in last 2 weeks	8.3 (45/545)	13.7 (14/102)	38.0 (38/100) *†	<0.0001
Child used bed net last night (%)	52.7 (286/543)	69.6 (71/102) *	73.3 (74/102) *	<0.0001
Chloroquine in plasma (%)	3.7 (20/545)	4.0 (4/101)	4.0 (4/101)	0.98

Numerical data are means (range) unless otherwise indicated, and compared by the non-parametric Kruskal Wallis and Mann Whitney U tests. Proportions were compared by χ^2 ^test or Fisher's exact test. ^a ^, *n *= 748; *, difference to Huye communities, *P *< 0.05; †, difference to Sovu health centre, *P *< 0.05

### Parasitological parameters

Overall, 16.7% of all 749 children were found by PCR to harbour *P. falciparum *, 11.7% had microscopically visible parasitaemia, and 5.5% malaria. All microscopically positive samples were also positive by PCR (including one *Plasmodium malariae *and two *Plasmodium ovale *mono-infections). The prevalences of *P. falciparum *infections detected by PCR (range, 16-21%) and of microscopically visible parasitaemia (range, 10%-17%) did not differ between the groups (Table 3). However, whereas only one quarter of community children with parasitaemia was classified as having malaria, this was the case in all children at the health centre and in most at the district hospital. Likewise, GMPDs were lower in community children as compared to health centre (*P *= 0.02) or, non-significantly, to district hospital (*P *= 0.40; Table 3). As for the non-falciparum parasites, *P. malariae *was rare in the community but reached 3% in the health centre (*P *= 0.01).

**Table 3:** Parasitological parameters in 749 children from southern highland Rwanda

Parameter	Huye communities	Sovu Health Centre	Kabutare District Hospital	*P *
No.	545	103	101	
Parasitaemia (%)	11.2	16.5	9.9	0.25
GMPD (parasites/μL; 95%CI)	1574 (913-2714)	7603 (2127-27185) *	5508 (701-43251)	0.04
MOI (mean, range)	2.05 (1-5)	1.95 (1-4)	1.92 (1-4)	0.92
Malaria (%)	2.9	16.5 *	7.9 *	<0.0001
*P. falciparum *infection, PCR (%)	16.1	21.4	14.9	0.37
*P. ovale *infection, PCR (%)	0.9	1.9	3.0	0.22
*P. malariae *infection, PCR (%)	0.2	2.9 *	2.0	0.006
Proportion of submicroscopic infections (%, n/n)	33.7 (31/92)	22.7 (5/22)	41.2 (7/17)	0.45
Child received artemether-lumefantrine in last 2 weeks	3.1	1.0	5.9	0.13

**Figure 3:**
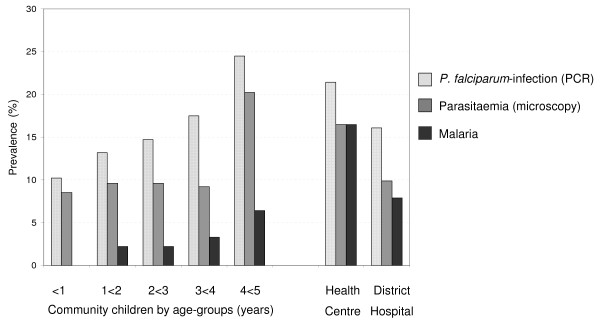
**Prevalences of *P. falciparum *infection (PCR), parasitaemia (microscopy) and malaria in 749 children from southern highland Rwanda**.

GMPD, geometric mean parasite density, and MOI, multiplicity of infection, are compared by the non-parametric Kruskal Wallis and Mann Whitney U tests. Proportions were compared by χ^2 ^test or Fisher's exact test. *, difference to Huye communities, *P *< 0.05;

In the villages, the prevalence of *P. falciparum *infection (PCR) ranged from 0 to 38.5% (*P *= 0.0002; Figure 2). The number of children in these communities allowed age-stratified analysis of parasitological parameters (numbers in age-groups: <1 year, 59; 1<2 years, 136; 2<3 years, 136; 3<4 years, 120; 4<5 years, 94). By χ^2 ^test for trend, the prevalences of *P. falciparum *-infection by PCR (*P *= 0.009), of microscopically visible parasitaemia (*P *= 0.03), and of malaria (*P *= 0.02) increased with age (Figure 3). Likewise, the proportion of asymptomatically infected children (PCR positive but no current or history of fever) among all infected children tended to decline with every year of age (100% (6/6), 88.8% (16/18), 85.0% (17/20), 81.0% (17/21), and 73.9% (17/23), *P *= 0.08). GMPDs (95% CIs) did not show a clear trend: in the above age groups, they were 308 (143-663), 1,374 (513-3,680), 1,162 (339-3,975), 3,491 (714-17,063), and 2,061 (781-5,437) parasites/μL, respectively (*P *= 0.29).

Multiplicity of infection (MOI) was successfully typed for 88.8% (111/125) of all *P. falciparum *isolates. MOI ranged from one to five (mean, 2.02; median, 2.0); 65% (72/111) of infections were polyclonal (MOI >1). MOI did not differ between community children and those attending health facilities (Table 3), did not correlate with age (months; Spearman's *r *= 0.14; *P *= 0.33; community children only, *r *= 0.19, *P *= 0.20) but was increased in microscopically visible parasitaemia as compared to submicroscopic infections (means, 2.15 *vs*. 1.67; *P *= 0.03), correlated positively with parasite density (*r *= 0.28, *P *= 0.006), and tended to be reduced in former artemether-lumefantrine (AL) recipients as compared to children without such reported intake (means, 1.54 *vs*. 2.08; *P *= 0.06).

### Factors associated with *P. falciparum *infection and malaria

Beyond age, a number of factors influenced the presence of infection or malaria. In explorative univariate analysis, socio-economic parameters (Table 2), MUAC, self-reported bed net use, previous AL treatment, and chloroquine in plasma were tested for association with *P. falciparum *infection. The odds of *P. falciparum *was found to increase with increasing age, decreasing MUAC, low educational level, absent father, absence of several household assets, a low family income, lacking use of a bed net, intake of AL within the preceding two weeks (median 7 days before; range, 1-14), and the presence of chloroquine in plasma. For multivariate analysis, univariately associated factors, adjusted for study sub-groups, were entered into a logistic regression model, and subjected to stepwise backward removal (Table 4). Independent predictors of *P. falciparum *infection included increasing age, health centre attendance, low MUAC, absence of cupboard, radio and bicycle, recent AL intake, and presence of chloroquine in plasma.

**Table 4:** Univariate and multivariate analysis of factors associated with *P. falciparum *infection (PCR)

Parameter	**No**.	Proportion infected (%)	Univariate analysis			Multivariate analysis		
				
			OR	95%CI	*P *	aOR	95%CI	*P *
Group								
Huye communities	545	16.1	1			1		
Health Centre	101	21.4	1.41	0.81-2.45	0.20	2.74	1.44-5.19	0.002
District Hospital	103	14.9	0.91	0.48-1.70	0.74	1.72	0.86-3.44	0.12
Age (months)	749	n.a.	1.02	1.01-1.03	0.001	1.03	1.01-1.04	<0.0001
MUAC (cm)	748	n.a.	0.88	0.79-0.99	0.03	0.83	072-0.95	0.008
Mother's education								
None	201	20.9	1					
Primary	504	16.1	0.72	0.47-1.12	0.13			
Secondary/tertiary	42	4.8	0.19	0.02-0.79	0.01			
Father's education								
None	235	23.0	1					
Primary	451	14.2	0.55	0.36-0.85	0.004			
Secondary/tertiary	51	7.8	0.29	0.07-0.84	0.01			
Father's occupation								
Farmer/labourer	611	16.9	1					
Else	91	8.8	0.48	0.21-1.05	0.05			
Died/left/prisoner	42	31.0	2.21	1.05-4.60	0.02			
Pipe-born water								
No	622	18.3	1					
Yes	122	7.4	0.35	0.16-0.75	0.003			
Cupboard								
No	633	18.3	1			1		
Yes	111	6.3	0.30	0.11-0.66	0.002	0.37	0.15-0.92	0.03
Radio								
No	363	23.7	1			1		
Yes	380	9.5	0.34	0.22-0.52	<0.0001	0.40	0.24-0.66	0.0003
Bicycle								
No	628	18.5	1			1		
Yes	116	6.0	0.28	0.11-0.63	0.0009	0.38	0.15-1.0	0.049
Household income								
>= 5000 RwF (median)	498	14.5	1					
< 5000 RwF	250	21.2	1.59	1.05-2.40	0.02			
Use of bed net								
No	315	21.0	1					
Yes	431	13.7	0.60	0.40-0.90	0.009			
Intake of AL, preceding 2 weeks								
No	725	15.4	1			1		
Yes	24	54.4	6.47	2.64-15.9	<0.0001	6.93	2.70-17.78	<0.0001
Chloroquine in plasma								
No	719	14.5	1			1		
Yes	28	67.9	12.48	5.19-32.1	<0.0001	17.18	6.84-43.16	<0.0001

n.a., not applicable; MUAC, mid upper arm circumference; RwF, Rwandan Francs; AL, artemether-lumefantrine; OR, odds ratio; aOR, adjusted OR derived from logistic regression including all parameters listed here and following stepwise backward removal of factors not associated in multivariate analysis.

For malaria, the same analysis produced the following independently associated factors (aOR (95% CI)): age (months, 1.02 (1.0-1.05), *P *= 0.04), absent father (6.53 (2.15-19.83), *P *= 0.0009), presence of radio (0.30 (0.13-0.65), *P *= 0.003), recent intake of AL (4.40 (1.24-15.57), *P *= 0.02), and chloroquine in plasma (4.87 (1.43-16.57), *P *= 0.01), adjusted for attendance at health centre (15.93 (6.47-39.26), *P *< 0.0001) or district hospital (9.16 (3.16-26.52), *P *< 0.0001). The role of village among community children lost significance in multivariate analysis (all, *P *> 0.05). In the above final models, self-reported bed net use showed no association with *P. falciparum *infection (aOR, 0.88 (0.56-1.38), *P *= 0.58) or malaria (aOR, 0.99 (0.47-2.10), *P *= 0.98).

Limiting multivariate analysis to community children produced basically the same results. However, household possessions of cupboard or bicycle lost significant association with *P. falciparum *infection. In multivariate analysis of current malaria in community children, chloroquine in plasma (prevalence, 3.7%) lost significant association, and that of age (months) became borderline significant (aOR, 1.03; 95%CI, 1.0-1.07; *P *= 0.07).

### Clinical manifestations

Because ordinal regression analyses revealed that *P. falciparum *infection (PCR) in the three groups had significantly differing effects on Hb (*r *= 0.6; standard error (SE) = 0.25; *P *= 0.02) and body temperature (*r *= 0.83; SE = 0.25; *P *= 0.001), the main analysis was performed for community children (Table 5). In these, anaemia (Hb<11 g/dL) was observed in 34% and fever in 3%. Parasitaemia was associated with a reduction in mean Hb of-2.2 g/dL, and, age-adjusted, eighteen-fold and five-fold increased odds of anaemia and fever, respectively. These effects were pronounced at increasing parasite density. However, even in submicroscopic infections (all afebrile), mean Hb was significantly reduced by-1.4 g/dL (Table 5).

**Table 5:** Manifestation of malaria in rural Huye subdistrict

		Fever			Anaemia (Hb < 11 g/dl)			Hb (g/dL)	
				
	**No**.	%	*P *	aOR (95%CI)	%	*P *	aOR (95%CI)	Mean	*P *
Parasitaemia									
Absent	484	2.3 (11/482)			28.1			11.5	
Present	61	11.5	0.002	4.8 (1.8-13.2)	82.0	<0.001	17.5 (8.4-36.4)	9.3	<0.0001
Parasite density									
None	484	2.3 (11/482)			28.1			11.5	
< 1000	26	7.7	0.1	3.4 (0.7-16.5)	65.4	0.0002	6.0 (2.5-14.4)	9.9	<0.0001
1000 < 10000	25	12.0	0.01	4.8 (1.2-18.9)	96.0	<0.0001	105.7 (13.8-812.5)	9.1	<0.0001
> = 10000	10	20.0	0.005	8.6 (1.6-46.9)	90.0	0.003	40.5 (4.9-336.7)	8.3	<0.0001
*P. falciparum *infection, PCR									
Absent	457	2.4 (11/455)			26.7			11.6	
Present	88	8.0	0.008	3.0 (1.1-8.1)	72.7	<0.0001	10.8 (6.1-18.9)	9.6	<0.0001
Staged infection									
None	453	2.4 (11/451)			26.5			11.6	
Submicroscopic	31	0	1	0 (0-∞)	51.6	0.004	4.0 (1.9-8.8)	10.2	0.0009
Microscopic	61	11.5	0.004	4.4 (1.6-12.2)	82.0	<0.0001	20.3 (9.6-42.6)	9.3	<0.0001

aOR, age-adjusted odds ratio. 95%CI, 95% confidence interval. Hb, haemoglobin concentration. Hb levels are compared by the non-parametric Mann Whitney U test.

In children attending the health centre, there were trends only of an association between anaemia and *P. falciparum *infection by PCR (age-adjusted OR (95% CI), 2.77 (0.90-8.55), *P *= 0.08) or microscopically visible parasitaemia (2.69 (0.80-9.0), *P *= 0.11). In contrast, at the district hospital, these figures yielded statistical significance (PCR, 4.62 (1.34-15.95), *P *= 0.02; microscopy, 10.3 (1.89-55.88), *P *= 0.007). For fever, the opposite was seen: Parasitaemia, e.g. detected by microscopy, increased the risk of fever nine times in the health centre (age-adjusted OR (95% CI), 8.88 (2.54-31.04), *P *= 0.0006) but showed no association in the hospital (1.27 (0.30-5.39), *P *= 0.74).

## Discussion

Malaria transmission in Rwanda varies widely. Traditionally, the central plateau (altitude 1,500 to 1,800 m) is considered as one of four distinct ecological zones with overall *P. falciparum *prevalence rates of 5% to 15% [17]. While transmission in Rwanda is regarded to be stable with seasonal peaks in the valleys and unstable (and potentially epidemic-prone) at higher altitude [18], a linear correlation between altitude and transmission would be over-simplified: in a recent tabulation of the years 2001-2007, endemicity at 1,600-2,000 m above sea level ranged from hypo-to holoendemic and annual malaria incidences (presumed and confirmed) from 2.4 to 20.4 per 1000 capita [17]. Likely, these figures have declined in recent years [4,7,8]. Data from the 2007-2008 Demographic and Health Survey (DHS) [19] indicate that in >4,600 children <5 years of age and sampled across Rwanda, 2.6% had malaria infection based on rapid *Plasmodium *lactate dehydrogenase tests. In the present study from the vicinity of Butare (altitude, 1,768 m), 11% and 16% of community children were infected with *P. falciparum *based on microscopy and PCR, respectively. Data from the 2010 Rwanda Health Management Information System indicate that 13.4% of patients attending health facilities in the Huye district had microscopically confirmed malaria. In the present study, this figure was 12.3%.

Given the scarcity of published community-based data, the reasons for the discrepancy in the prevalence of infection reported in the DHS and observed in the present study are difficult to appraise. Higher sensitivity of PCR as compared to microscopy or rapid test devices [20,21] may partially be involved. Selection bias during recruitment at home, e.g., due to preferential presentation by the parents of children with (a recent history of) sickness cannot completely be excluded. However, recruitment teams were instructed to select children from households randomly and into pre-defined age strata. Also, most infections in the communities were asymptomatic. One limitation of the present study is its cross-sectional nature by which e.g., seasonal fluctuations are not reproduced. Geographical variation of infection between villages was evident (Figure 2) but attempts to relate this to e.g. altitude or proximity to a water stream, failed. Likely involved, most villages comprise homesteads scattered in the hills rather than agglomerated settlements [22]. Other parameters in the present study, e.g. self-reported bed net use, socio-economic factors, anaemia, were largely in the reported range [4,19,22]. Thus, the present data are not representative for the central plateau, let alone Rwanda, but rather provide a detailed and up-to-date picture of *P. falciparum *infection in southern highland communities and in health facilities serving this population. In contrast, routine health facility based surveillance has clear limitations in providing complete or representative data on e.g., malaria in the community, also because patients lacking access or choosing alternatives are not registered [23]. A low health insurance coverage (43%) in the communities may have deterred parents from seeking formal health care. Such, in turn, could have lead to an over-estimation of disease burden at the community level as compared to the end of the year when more have paid their fees. Nevertheless, only 20% of African children with suspicion of malaria are considered to come to the attention of any formal health system [24], a figure that might have improved in recent years [4]. Community-based surveys, despite their local limitations, thus provide essential information, also for control campaign monitoring [10,11].

In the communities, infection prevalence increased from 10% to almost 25% at four years of age, which was not accompanied by a decline in parasite density with age or increase in MOI. The additional age-dependent increase in malaria and trend for declining asymptomatic infections indicate that semi-immunity did not develop to the extent observed in highly endemic areas [25-27]. In line with this, *P. falciparum *infection including submicroscopic ones had an impact on Hb levels, which exceeds the one commonly seen in children in high-endemicity areas [27-29]. On the other hand, only a quarter of parasitaemic children had malaria, suggesting a majority of asymptomatic infections. Irrespective of the erratic nature of fever in the definition of malaria, the presence of (usually undetected) asymptomatic *P. falciparum *infections has important implications for malaria control in highland areas. In two sites in highland Kenya, both, high and low levels of asymptomatic *P. falciparum *infections have been observed among children and adults [30,31]. Studies from Ghana and Sudan indicate that asymptomatic infections can persist for a year or longer [32,33]; in highland Kenya the median duration in children aged 5-9 years was five months [31]. Gametocyte carriage was not consistently assessed in the present study but appeared to be low. Nevertheless, individuals with low level, long-lasting, and asymptomatic infections form a major reservoir for transmission [21,34,35]. In situations of increased rainfall, higher temperatures, or changed land use such asymptomatic infections may give rise to epidemics which have increased in frequency and intensity in East Africa during the last two decades [36-39]. Targeted antimalarial treatment even of asymptomatic children may thus be a justifiable part of malaria control in highland areas. However, the differing findings on the level of asymptomatic parasite carriage in the present and the two Kenyan studies [30,31] illustrate that results may not be readily extrapolated.

At the health facilities, roughly half of the children had respiratory tract infections or gastrointestinal problems. Every sixth child at the health centre had malaria and every fifth was *P. falciparum *infected. Irrespective of the better socio-economic status of the patients' families as compared to the communities (Table 2) this indicates that malaria is among the top three reasons to seek primary health care in this area. At eight percent prevalence, malaria was of lesser importance at the district hospital which receives referrals from several health centres and patients bypassing the referral system by self-paying. There, 38% of the patients were reportedly pre-treated including 6% with AL. The validity of (malaria) treatment histories frequently is questionable [40], and no data on the dose and duration of treatment were collected in the present study. Nevertheless, the finding that recent AL treatment was positively associated with current malaria is remarkable. This is suggestive of recurrence of parasitaemia following treatment. In fact, drug resistance markers associated with reappearing parasitaemia following AL treatment tended to be increased in these infections (Zeile *et al *, unpublished observations). Latest cure rates of AL in Rwanda from 2006 have been reported as 97% [41]. Nevertheless, against the background of intense AL drug pressure in Rwanda in recent years, this finding underlines the necessity of the upcoming re-evaluation of the drug's efficacy in this country.

Intake of chloroquine was stated by none of the respondents but the drug was present in plasma in 1.4% and 15.4% of non-infected and infected children, respectively. With the assay applied, chloroquine intake can be detected for several weeks, depending on the dose; cross-reactivity with amodiaquine is negligible [16]. Likely, the finding of an increased infection prevalence in chloroquine positive children reflects the combination of previous home-treatment and persisting or recrudescent parasites due to intense chloroquine resistance which is prevalent in Rwanda [42].

Among the age-adjusted risk factors for *P. falciparum *infection was a decreasing MUAC. This crude proxy parameter for malnutrition was, however, not associated with malaria itself. Chronic malnutrition affects every second child in Rwanda [19] and compromises anti-pathogen immunity [43]. Lacking effect on malaria morbidity as observed in the present study corresponds with previous findings [29,44] but contrasts with others [28,45,46]. Possibly, the differential effect on infection and malaria depends on the specific yet unknown type of malnutrition in the study area. Considering the modifiable nature of this risk factor and Rwanda's recently renewed commitment to fight malnutrition, more research into this field is needed.

At variable statistical significance, several parameters reflecting low socio-economic status were associated with increased risks of infection. Remarkably, possession of a radio or a bicycle were independently associated predictors of reduced *P. falciparum *prevalence. This may reflect increased access to malaria-related information, improved awareness and increased usage of curative services. Given the latter is true, this points to accomplishable ways of reducing *P. falciparum *infection in the area, i.e. health communication and education.

Lastly, although self-reported bed net usage was in the previously observed range [4,19,22], the detectable impact was modest and non-significant in multivariate analysis. This finding points to deficits in an established mean of malaria prevention the efficacy of which has been confirmed in many studies [4,7,10,11]. The reasons may be diverse and rather involve caregivers' beliefs about causation and vulnerability as well as obstacles in translating knowledge into behaviour than insecticide resistance [47-49]. Nevertheless, these actual reasons need to be assessed at the community-level and subsequent campaigns should address potential obstacles to promote consistent and correct use.

## Conclusions

In this community and facility based survey on malariologic parameters in southern highland Rwanda, *P. falciparum *infection was observed in one out of six children under five years of age, without much variation between community and health facilities. While facility-based, most infections were symptomatic, the opposite was seen in the communities. These seemingly asymptomatic infections greatly contributed to anaemia and form an unrecognized source of transmission in the epidemic-prone highland area. Improved nutrition, identification and elimination of causes of low bed net effectiveness, and reinforced health education are promising and tangible measures to further reduce *P. falciparum *in this area of Rwanda. In parallel, community-based surveillance of malaria should be included to monitor the progress of malaria control.

## Competing interests

The authors declare that they have no competing interests.

## Authors' contributions

JBG, FPM, AM, and GH designed the study. JBG, CSt, CSh, NCA, CHL, and CK were responsible for patient recruitment, clinical and laboratory examinations, and logistics. IZ did the PCR analyses, TEA the ELISA assays, and ID, JBG, AU and FPM the statistical analyses. JBG and FPM wrote the paper with major contributions of the other authors. All authors read and approved the final manuscript.
